# Supplementary material for: Application of Lacunarity for Quantification of Single Molecule Localization Microscopy Images
Source: Cells. 2022 Oct 2;11(19):3105. doi: 10.3390/cells11193105 (PMC9562870; doi:10.3390/cells11193105)
Supplement: Supplementary file 1 [file cells-11-03105-s001.zip › cells-1910353-supplementary.pdf]

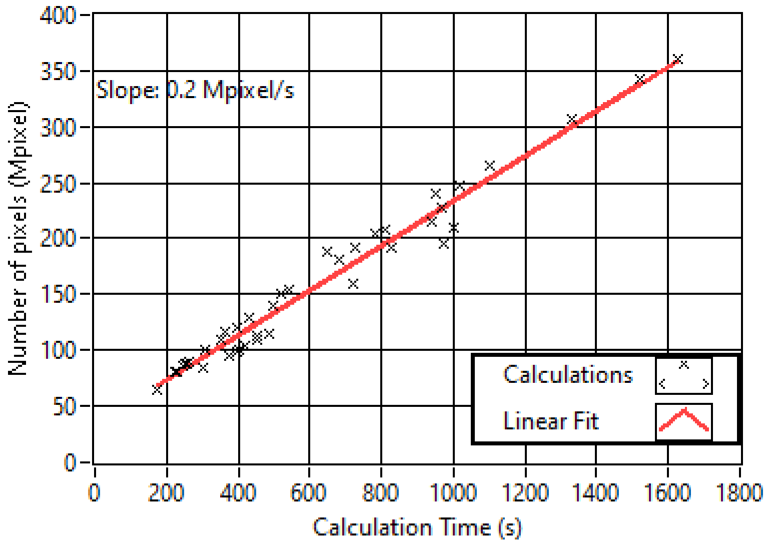

Figure S1. Size of each evaluated dSTORM image in megapixels plotted against the calculation time in seconds. The calculations were carried out on an AMD Ryzen 9 3900X processor at stock with simultaneous multithreading (SMT) enabled using 32 GBs of dual rank, dual channel, overclocked DDR4 3600 memory with 16-16-16-39 primary timings in clock cycles in UCLK=MEMCLK mode. All data were stored on a PCIe 4.0 Samsung 980 Pro 1 TB NVMe SSD. The code was written to utalize 12 out of the 24 threads of the CPU. The operating was Windows 10.

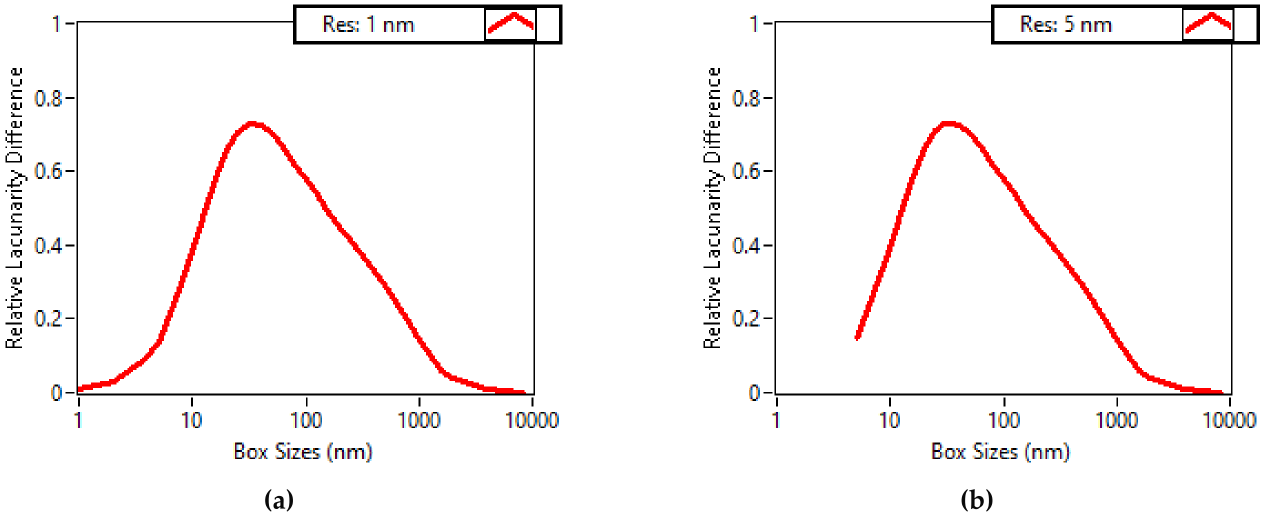

Figure S2. Lacunarity difference curves of the same dataset using different step sizes. Using a step size of 1 nm (**a**) takes 341.32 seconds, while using 5 nm stepsize (**b**) only takes 2.53 seconds. Using larger step sizes has the advantage of far shorter runtime, however we lose the information on box sizes smaller than the step size. The remaining points on the 5 nm step size curve show less than 0.5% difference from the 1 nm step size curve.

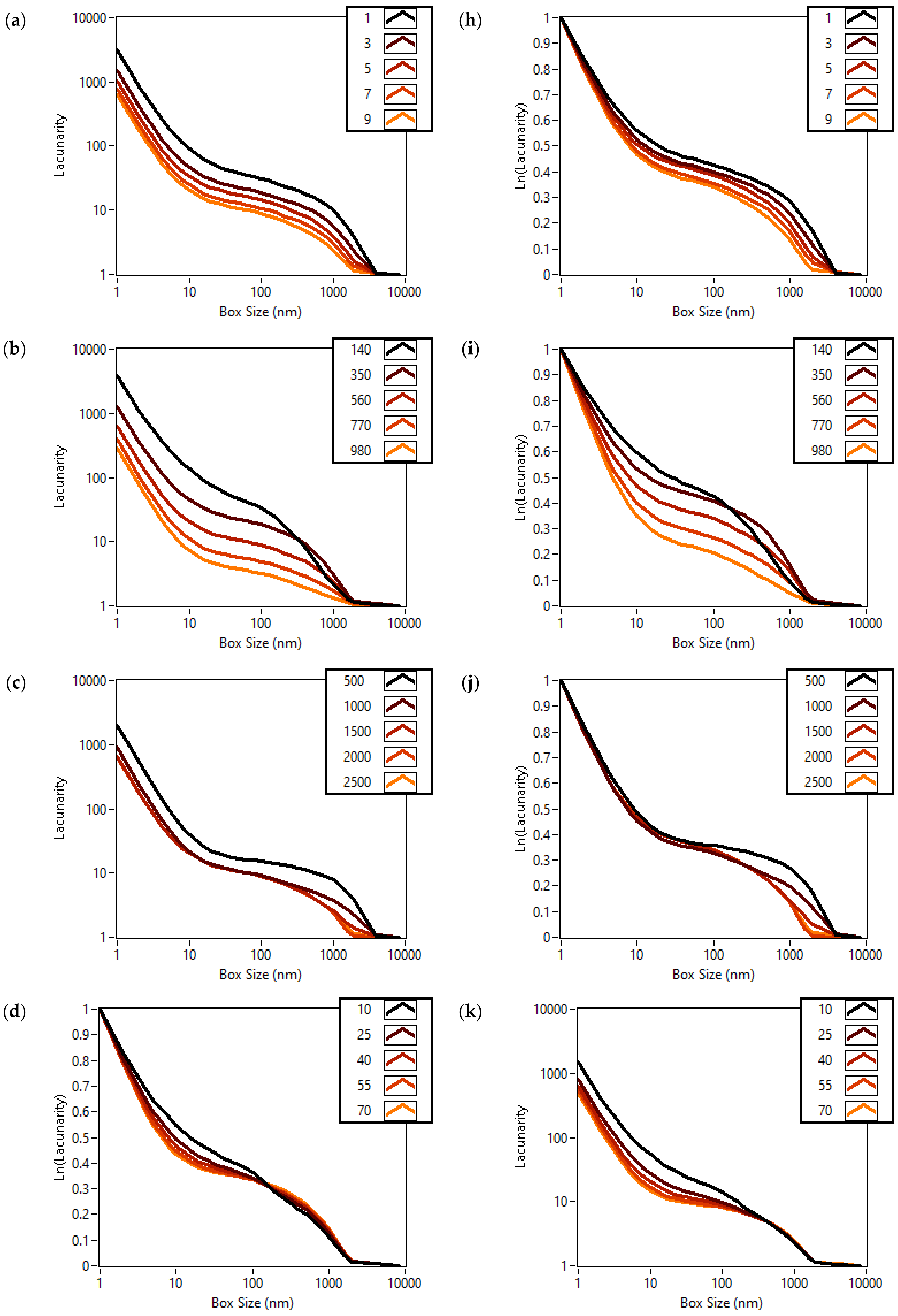
(a)
Lacunarity
Box Size (nm)
1
3
5
7
9
(h)
Ln(Lacunarity)
Box Size (nm)
1
3
5
7
9
(b)
Lacunarity
Box Size (nm)
140
350
560
770
980
(i)
Ln(Lacunarity)
Box Size (nm)
140
350
560
770
980
(c)
Lacunarity
Box Size (nm)
500
1000
1500
2000
2500
(j)
Ln(Lacunarity)
Box Size (nm)
500
1000
1500
2000
2500
(d)
Ln(Lacunarity)
Box Size (nm)
10
25
40
55
70
(k)
Lacunarity
Box Size (nm)
10
25
40
55
70

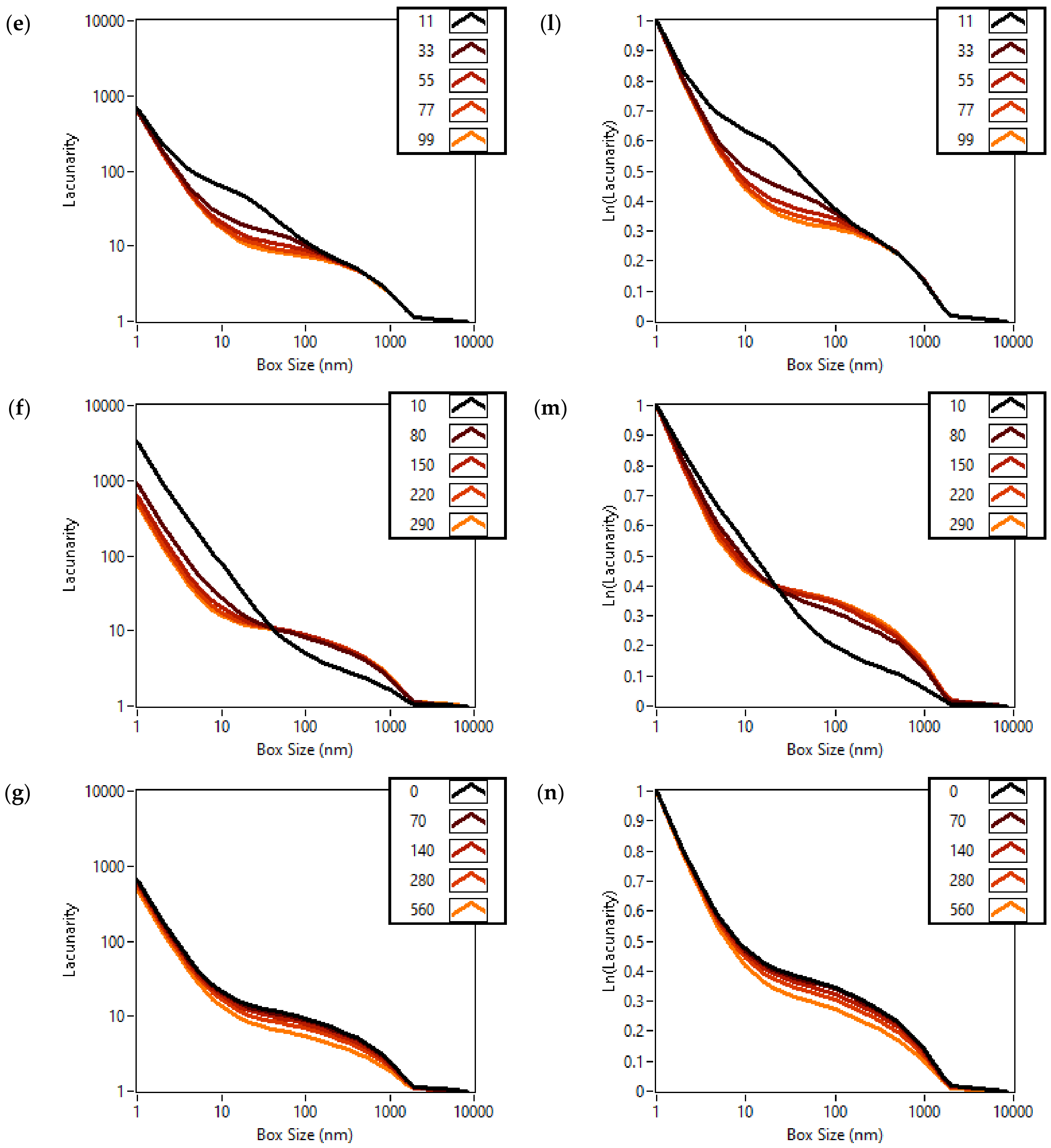

Figure S3. Lacunarity curves and conventionally normalized lacunarity curves of the TestSTORM simulations. Although conventional normalization is mathematically incorrect, it can help visualization. The lacunarity curves can be seen in images a-g and the normalized curves can be seen in images h-n. The five studied settings for each simulation in order are cluster number (**a**,**h**), cluster size in nanometers (**b**,**i**), cluster distance in nanometers (**c**,**j**), nanofocus density in foci per micrometer squared (**d**,**k**), nanofocus size in nanometers (**e**,**l**), localizations per nanofocus (**f**,**m**) and non-specific localizations in localizations per micrometers cubed (**g**,**n**).

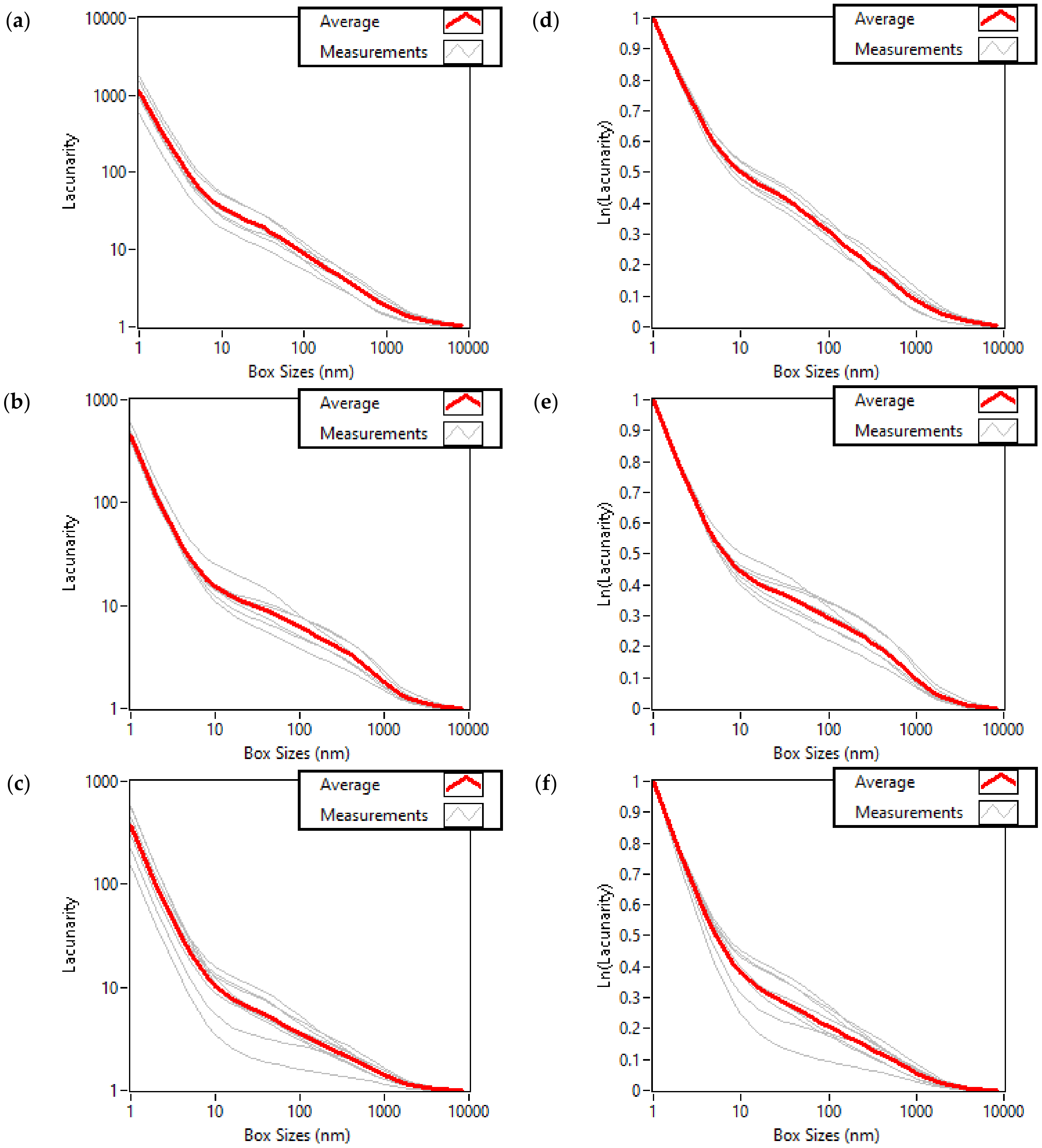

Figure S4. Lacunarity curves and conventionally normalized lacunarity curves of the radiation treated cells grouped by exposure. The nuclei were observed 30 minutes after being subjected to the following radiation doses: 0 Gy (**a**,**d**) 2 Gy (**b**,**e**) and 5 Gy (**c**,**f**).

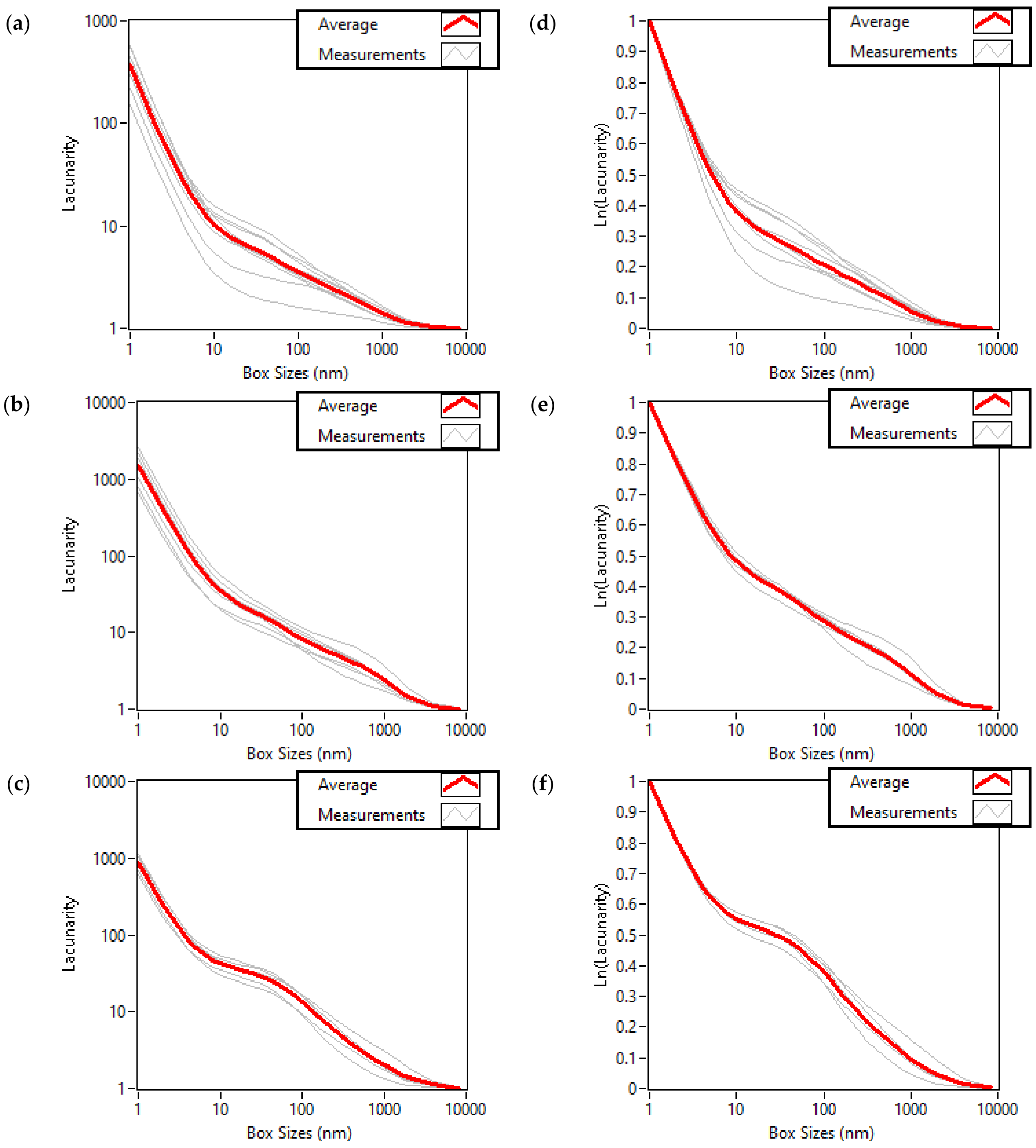

Figure S5. Lacunarity curves and conventionally normalized lacunarity curves of the radiaton treated cells grouped by time after treatment. The nuclei were subjected to a 5 Gy radiation dose and observed 30 minutes (**a**,**d**) 24 hours (**b**,**e**) and 72 hours after treatment (**c**,**f**).

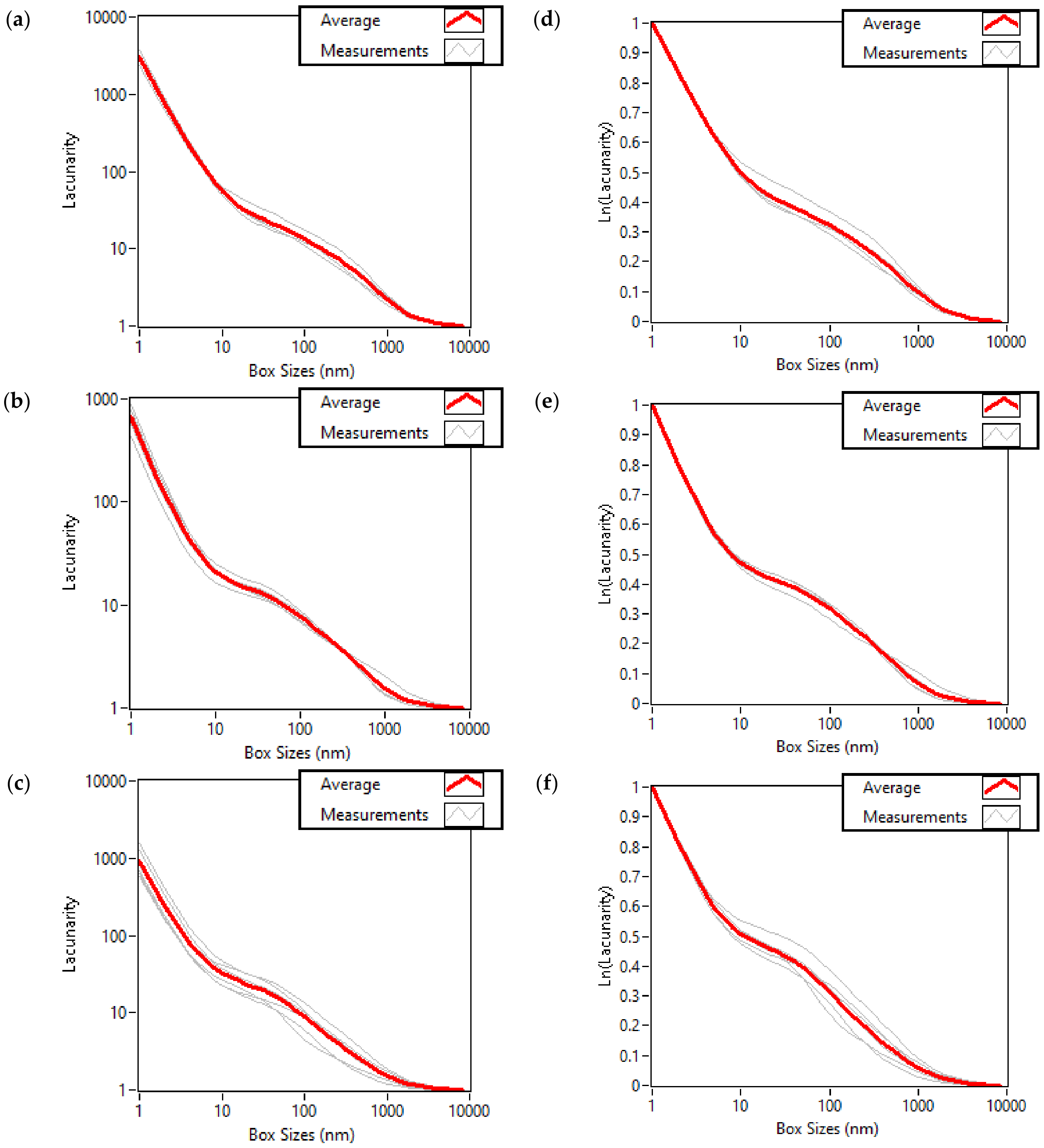

Figure S6. Lacunarity curves and conventionally normalized lacunarity curves of the chemically treated cells grouped by treatment. Untreated U2OS (**a**,**d**) neocarzinostatin treated U2OS (**b**,**e**) and 4-hydroxytamoxifen treated DIvA (**c**,**f**).
